# Supplementary material for: Coding of latent variables in sensory, parietal, and frontal cortices during closed-loop virtual navigation
Source: eLife. 2022 Oct 25;11:e80280. doi: 10.7554/eLife.80280 (PMC9668339; doi:10.7554/eLife.80280)
Supplement: MDAR checklist [file elife-80280-mdarchecklist1.zip › Figure2 supplement8.pdf]

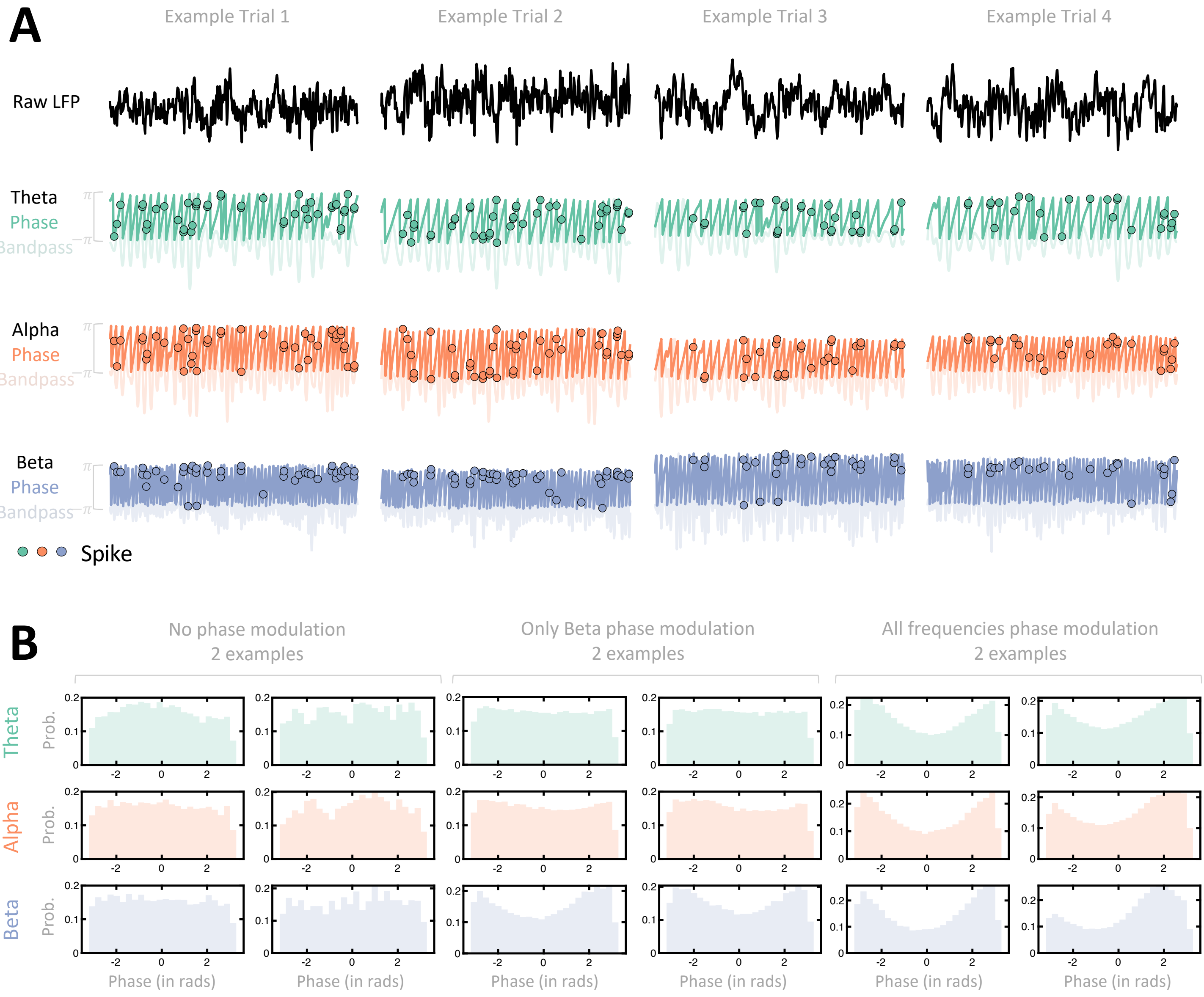

A
Example Trial 1
Example Trial 2
Example Trial 3
Example Trial 4
Raw LFP
Theta
Phase
Bandpass
Alpha
Phase
Bandpass
Beta
Phase
Bandpass
Spike
B
No phase modulation
2 examples
Only Beta phase modulation
2 examples
All frequencies phase modulation
2 examples
Theta
Alpha
Beta
Prob.
Phase (in rads)
