# Supplementary figures and images for: Coding of latent variables in sensory, parietal, and frontal cortices during closed-loop virtual navigation
Source: eLife. 2022 Oct 25;11:e80280. doi: 10.7554/eLife.80280 (PMC9668339; doi:10.7554/eLife.80280)

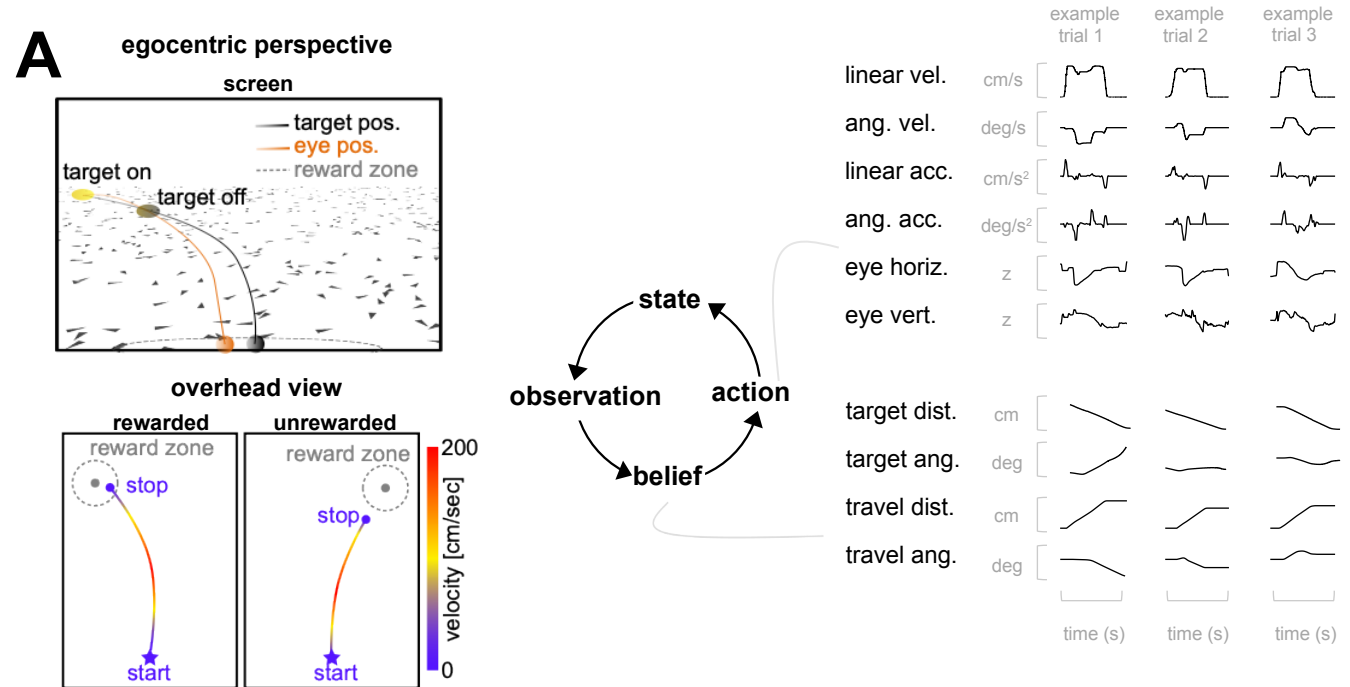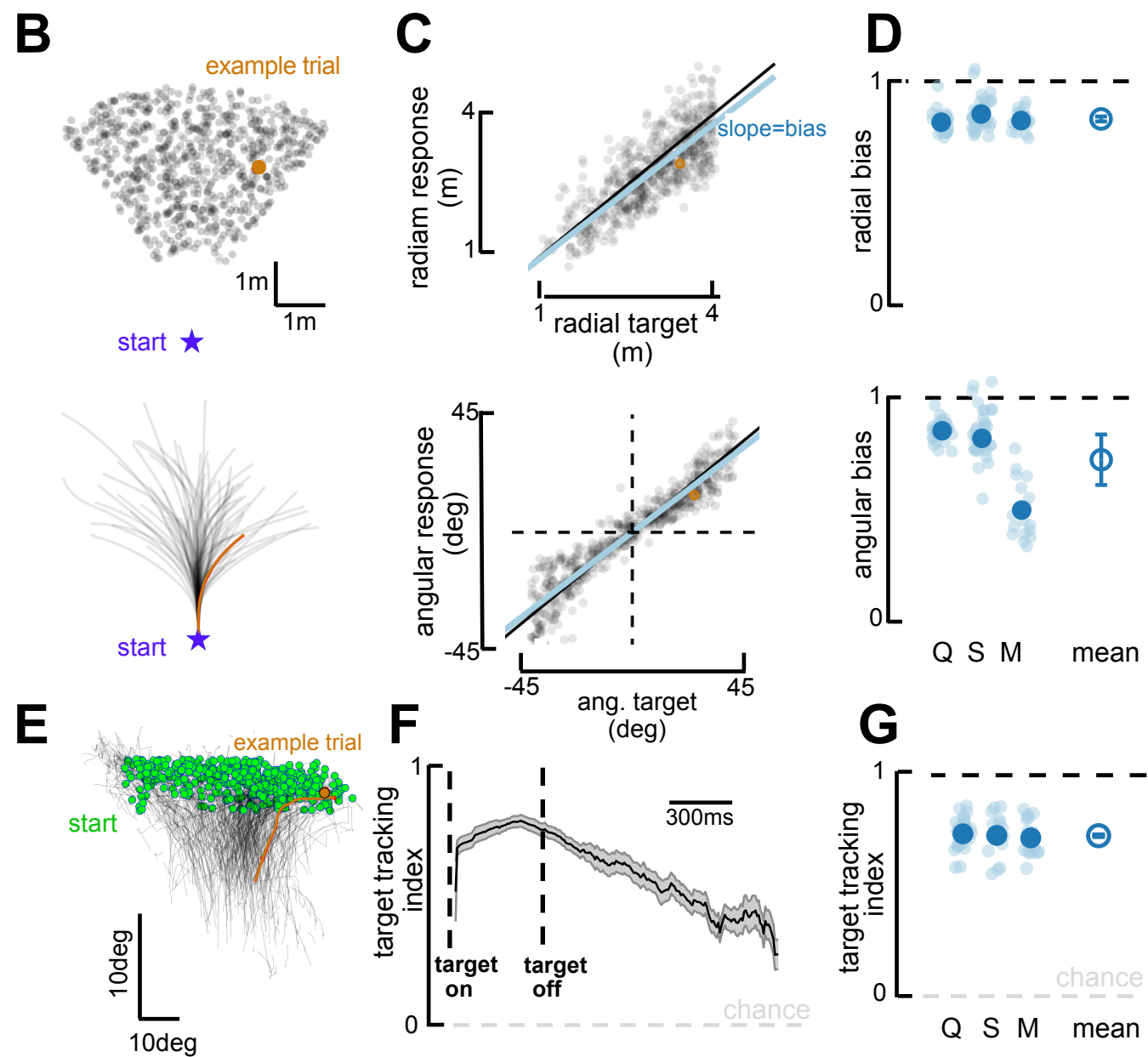

Supplement: MDAR checklist [file elife-80280-mdarchecklist1.zip › Figure1.pdf]

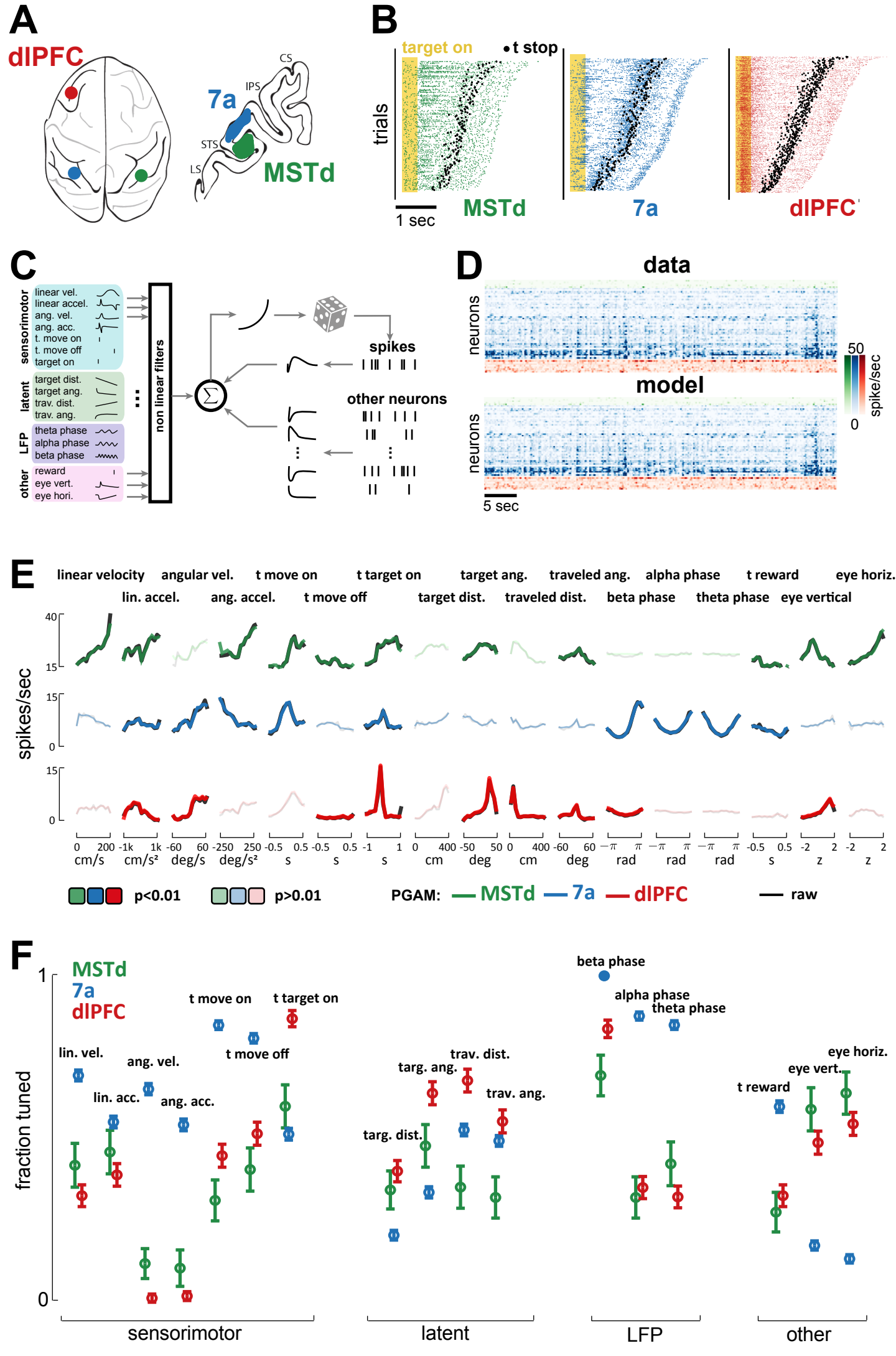

Supplement: MDAR checklist [file elife-80280-mdarchecklist1.zip › Figure2.pdf]

# Monkey S

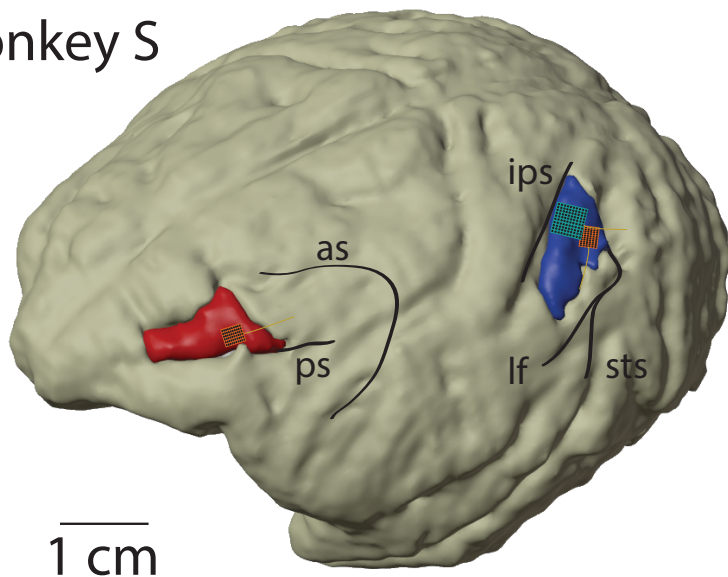

● Monkey S ● Monkey Q ● Monkey M

9/46

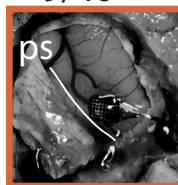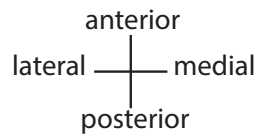

Area 7a

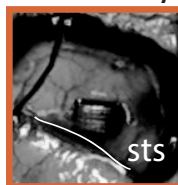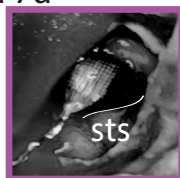

1 cm

MSTd  
7a  
dIPFC

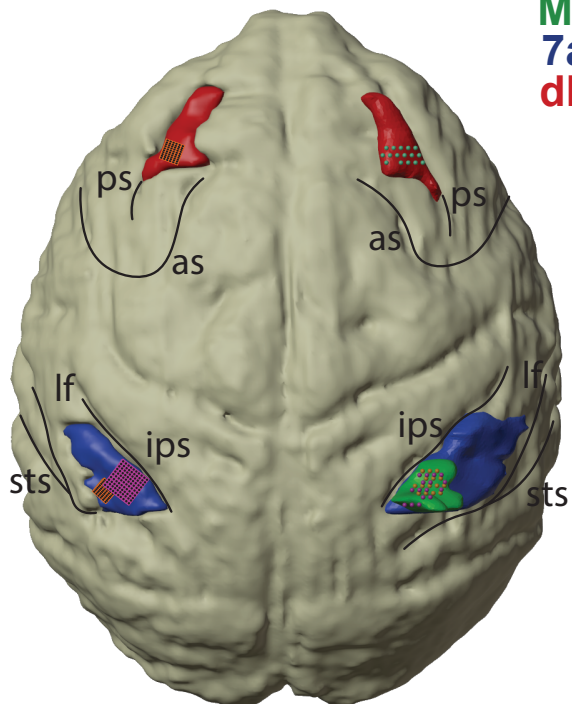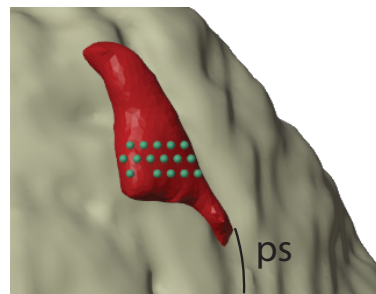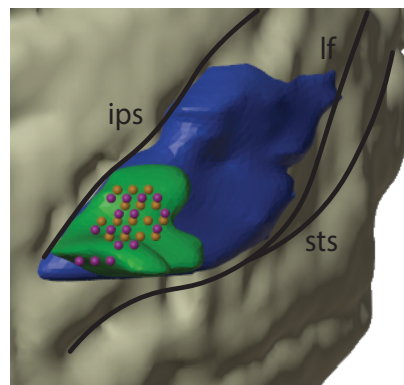

Supplement: MDAR checklist [file elife-80280-mdarchecklist1.zip › Figure2 supplement1.pdf]

**A**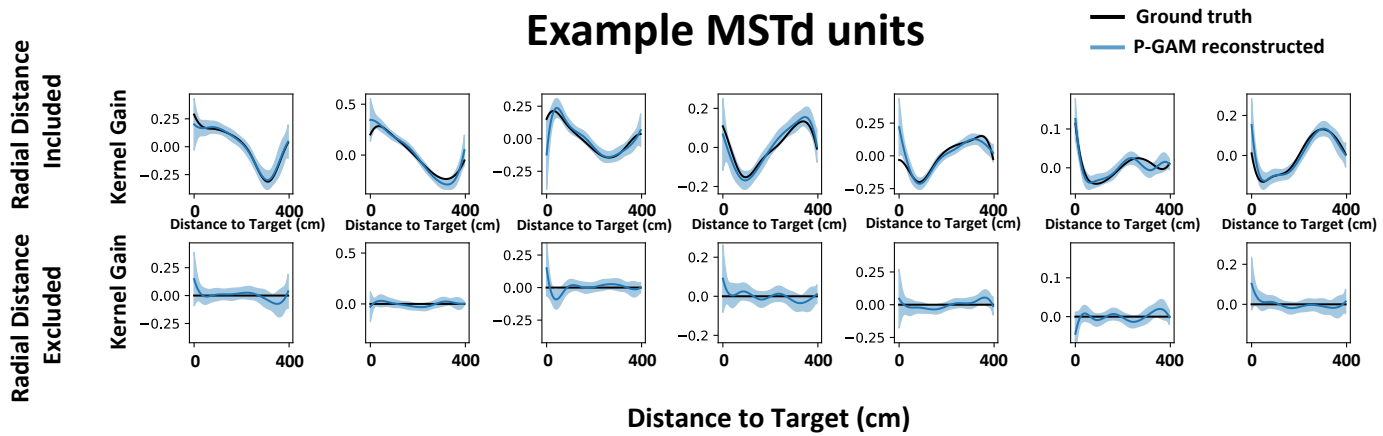**B**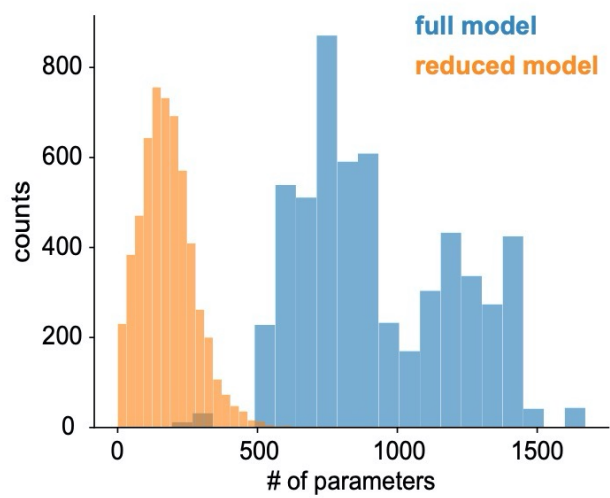**C**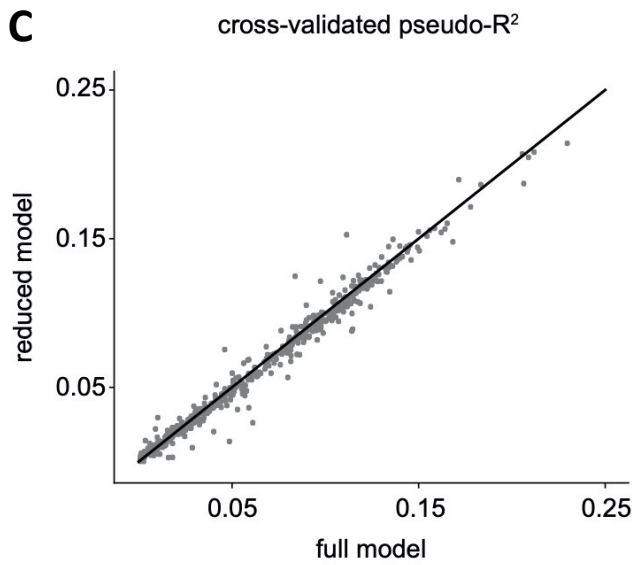

Supplement: MDAR checklist [file elife-80280-mdarchecklist1.zip › Figure2 supplement2.pdf]

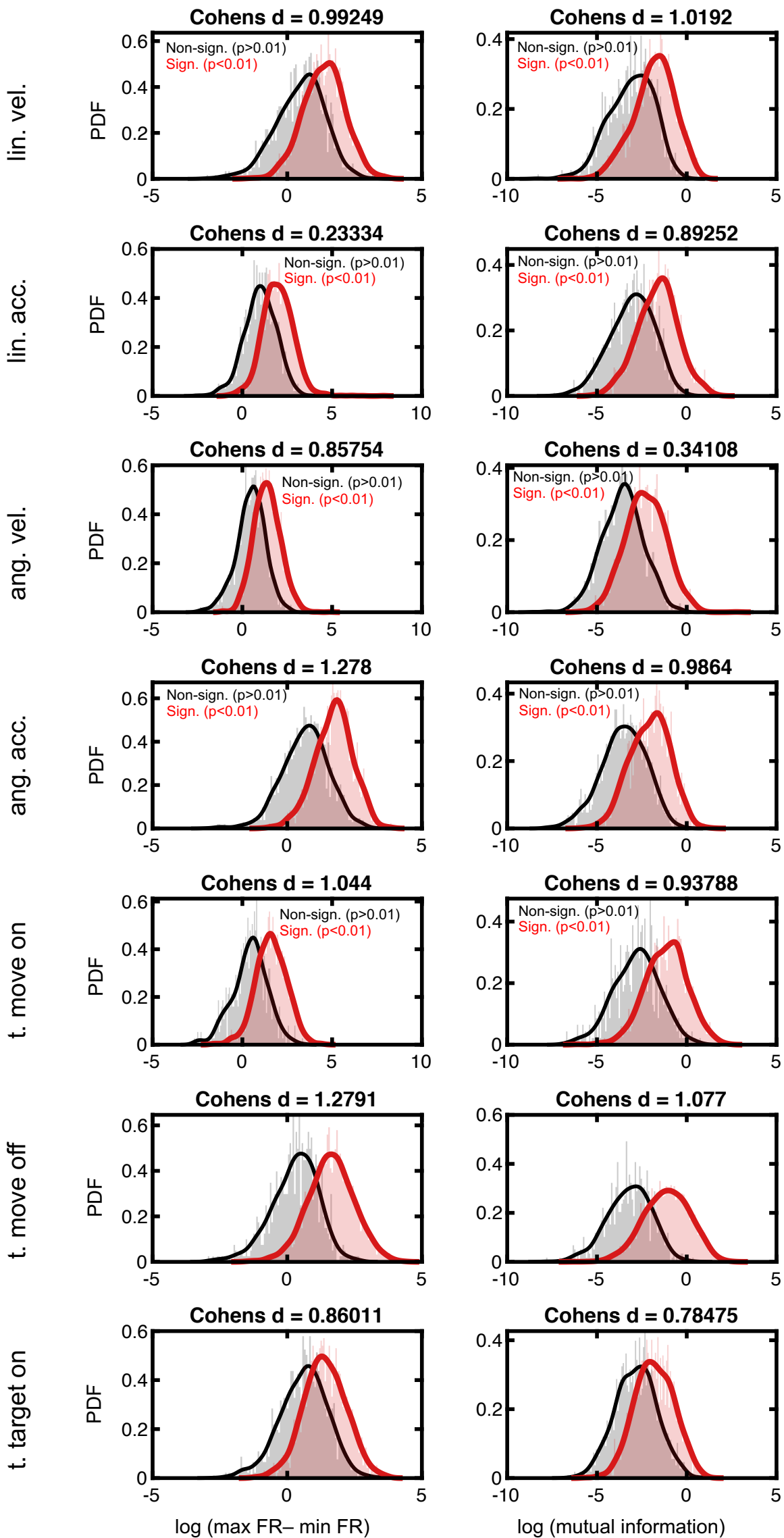

Supplement: MDAR checklist [file elife-80280-mdarchecklist1.zip › Figure2 supplement3.pdf]

targ. dist.

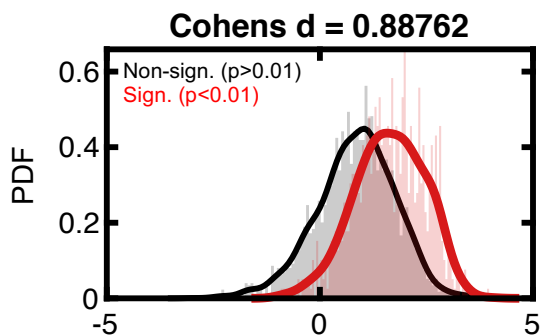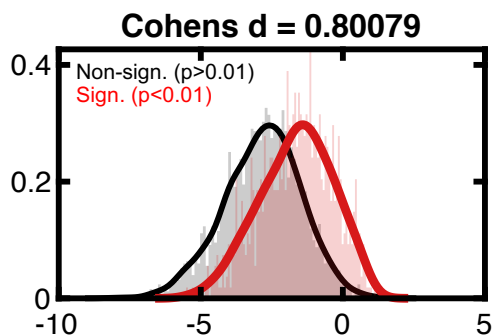

targ. ang.

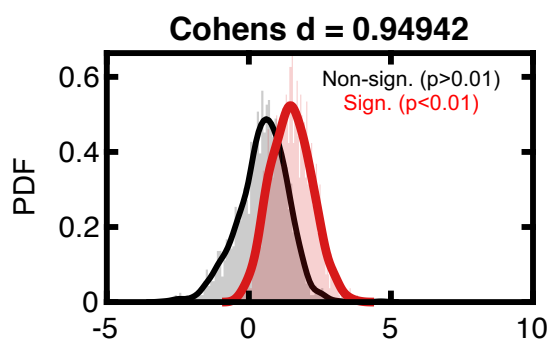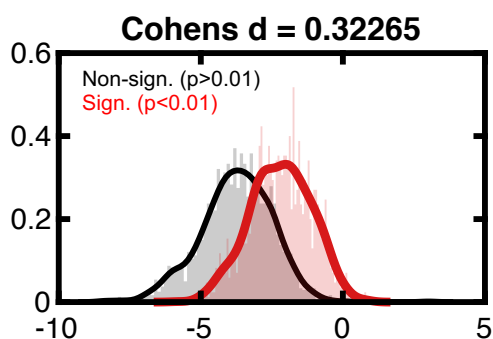

trav. dist.

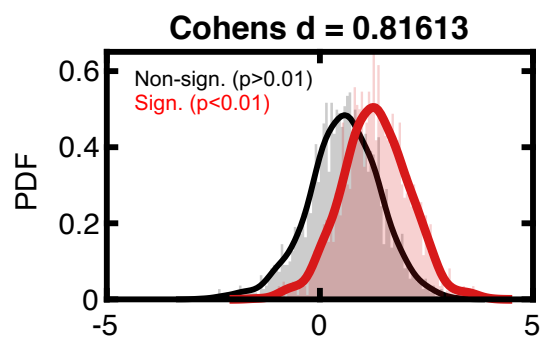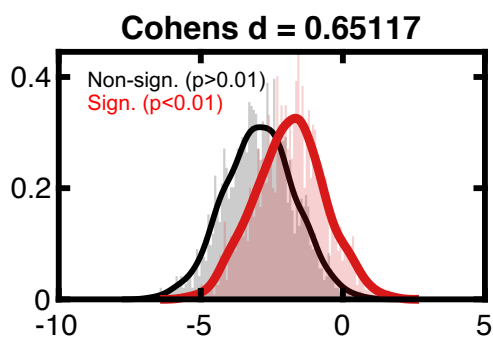

trav. ang.

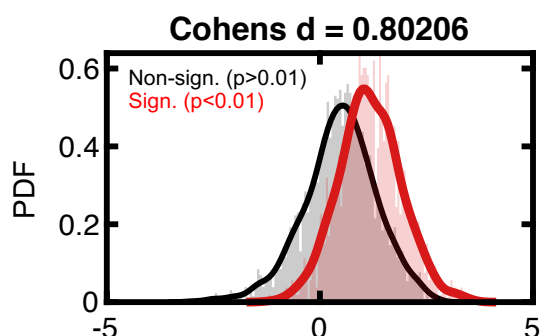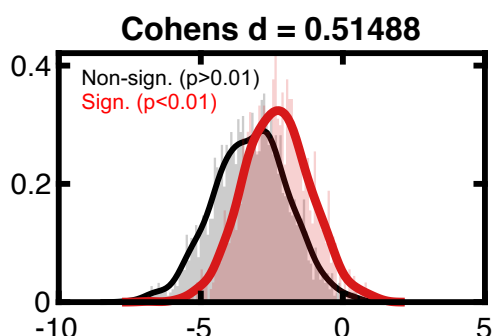

log (max FR– min FR)

log (mutual information)

Supplement: MDAR checklist [file elife-80280-mdarchecklist1.zip › Figure2 supplement4.pdf]

theta ph.

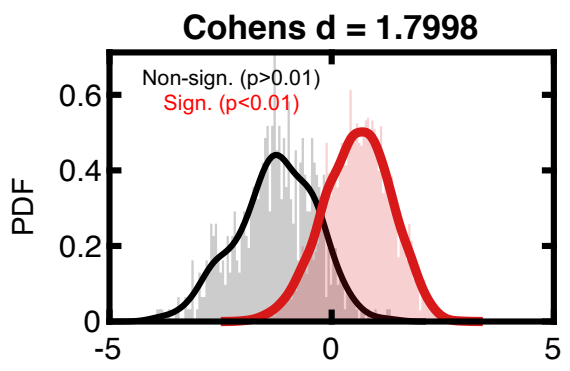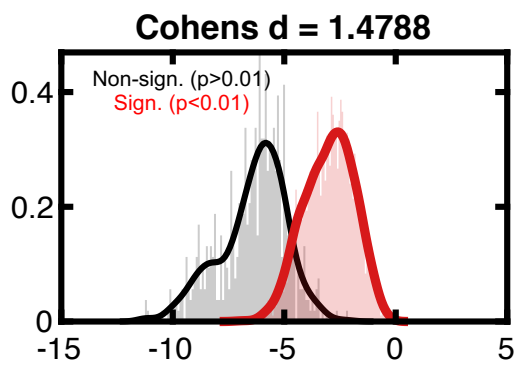

alpha ph.

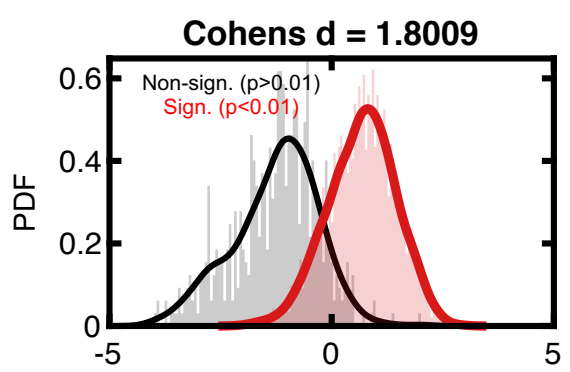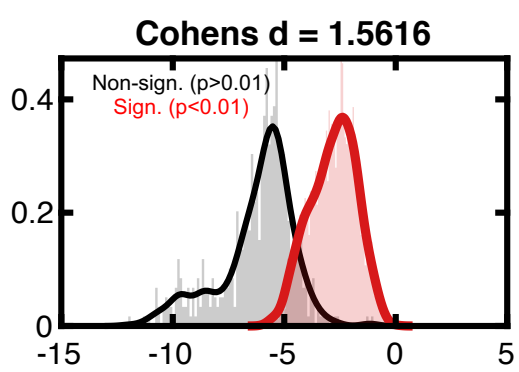

beta ph.

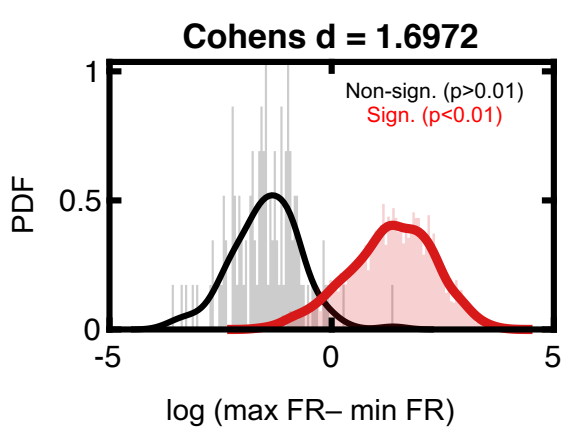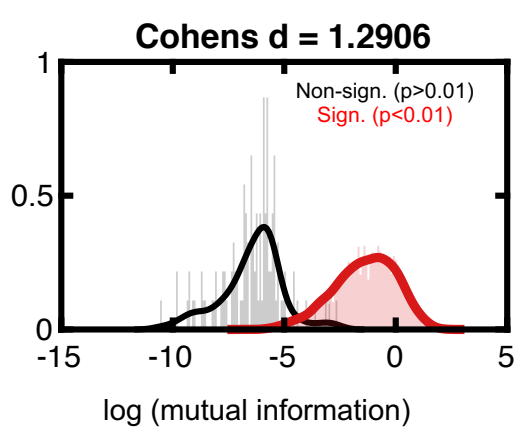

Supplement: MDAR checklist [file elife-80280-mdarchecklist1.zip › Figure2 supplement5.pdf]

t. reward.

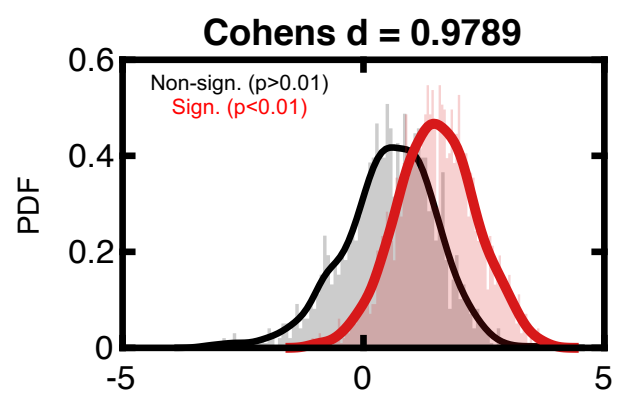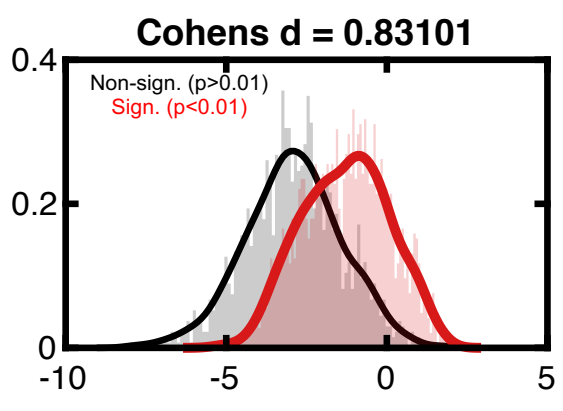

eye vert.

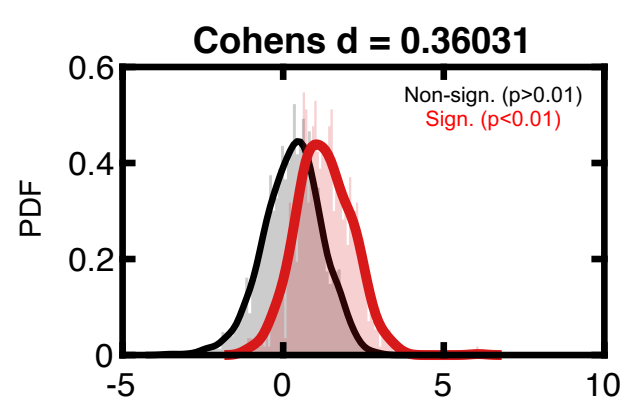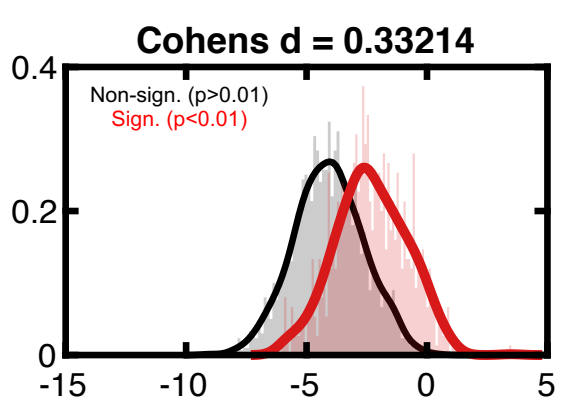

eye horz.

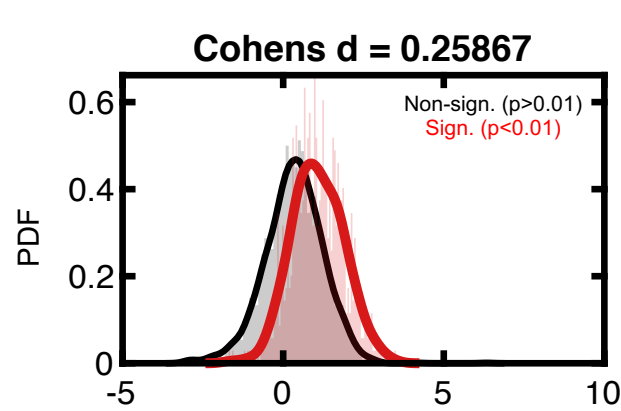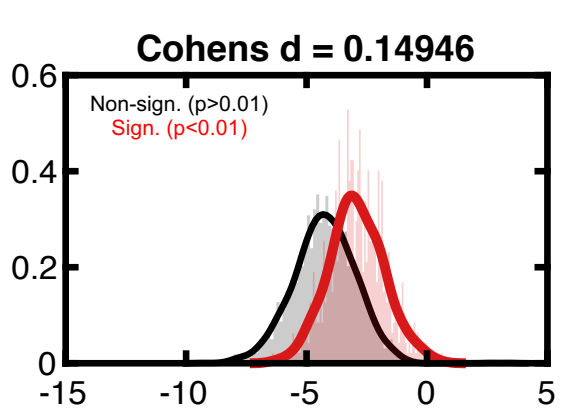

log (max FR– min FR)

log (mutual information)

Supplement: MDAR checklist [file elife-80280-mdarchecklist1.zip › Figure2 supplement6.pdf]

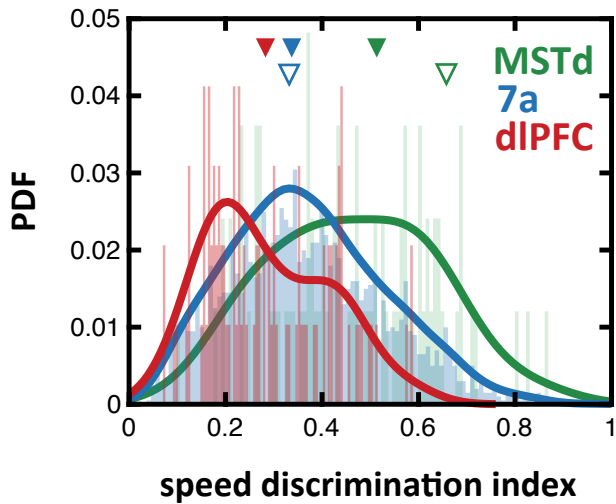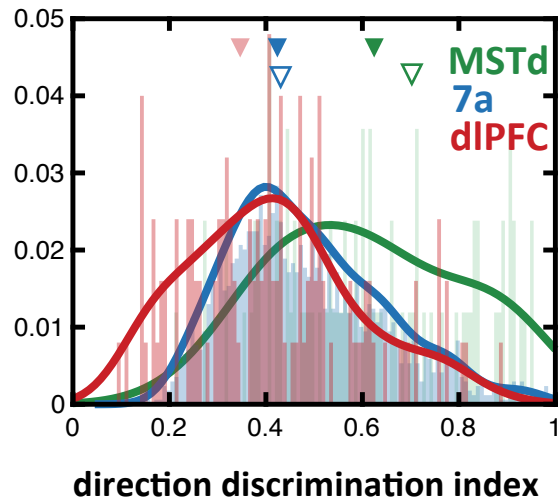

Supplement: MDAR checklist [file elife-80280-mdarchecklist1.zip › Figure2 supplement7.pdf]

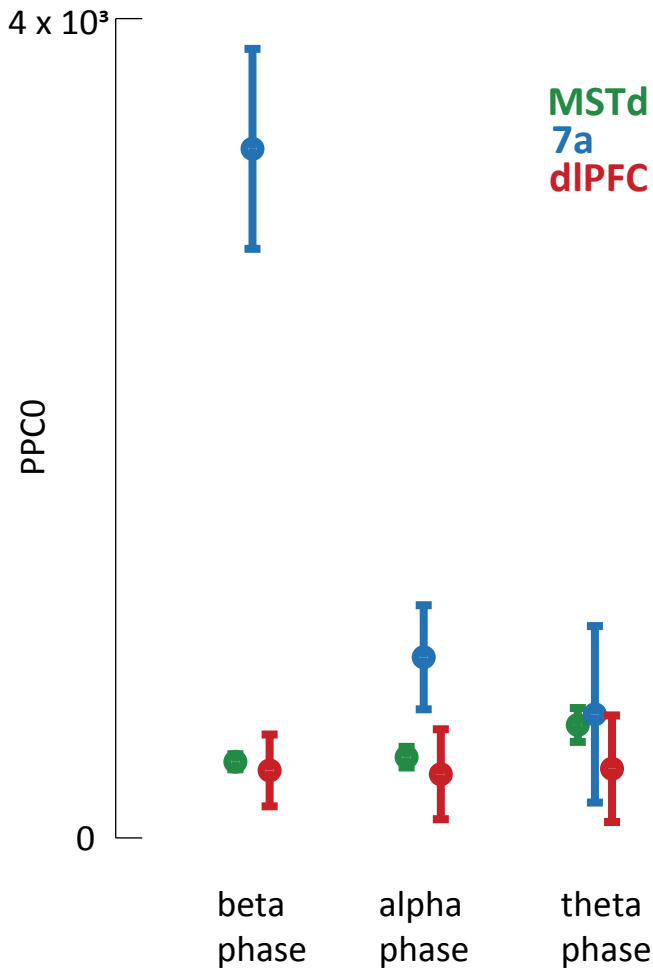

Supplement: MDAR checklist [file elife-80280-mdarchecklist1.zip › Figure2 supplement9.pdf]

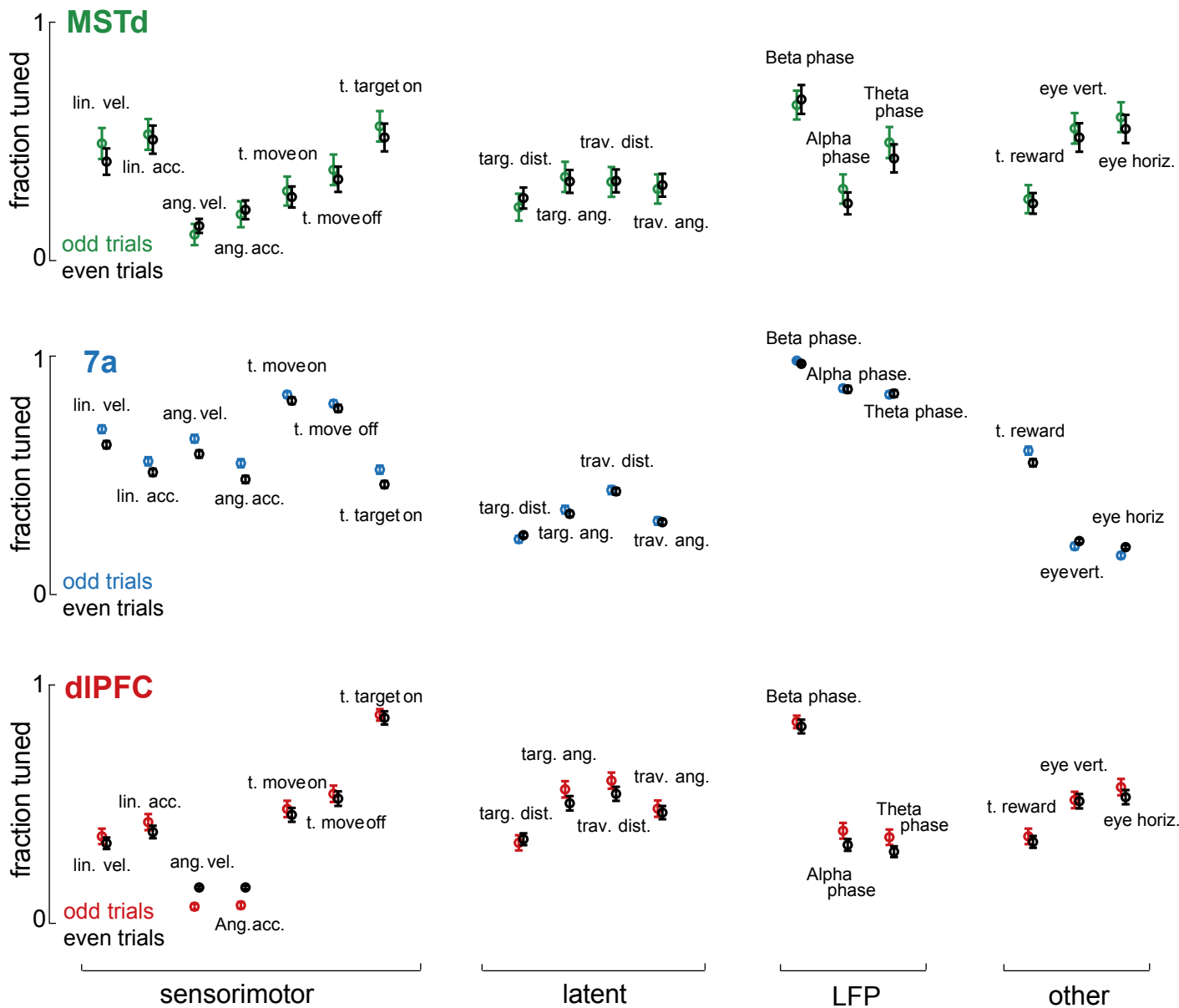

Supplement: MDAR checklist [file elife-80280-mdarchecklist1.zip › Figure2 supplement10.pdf]

**A**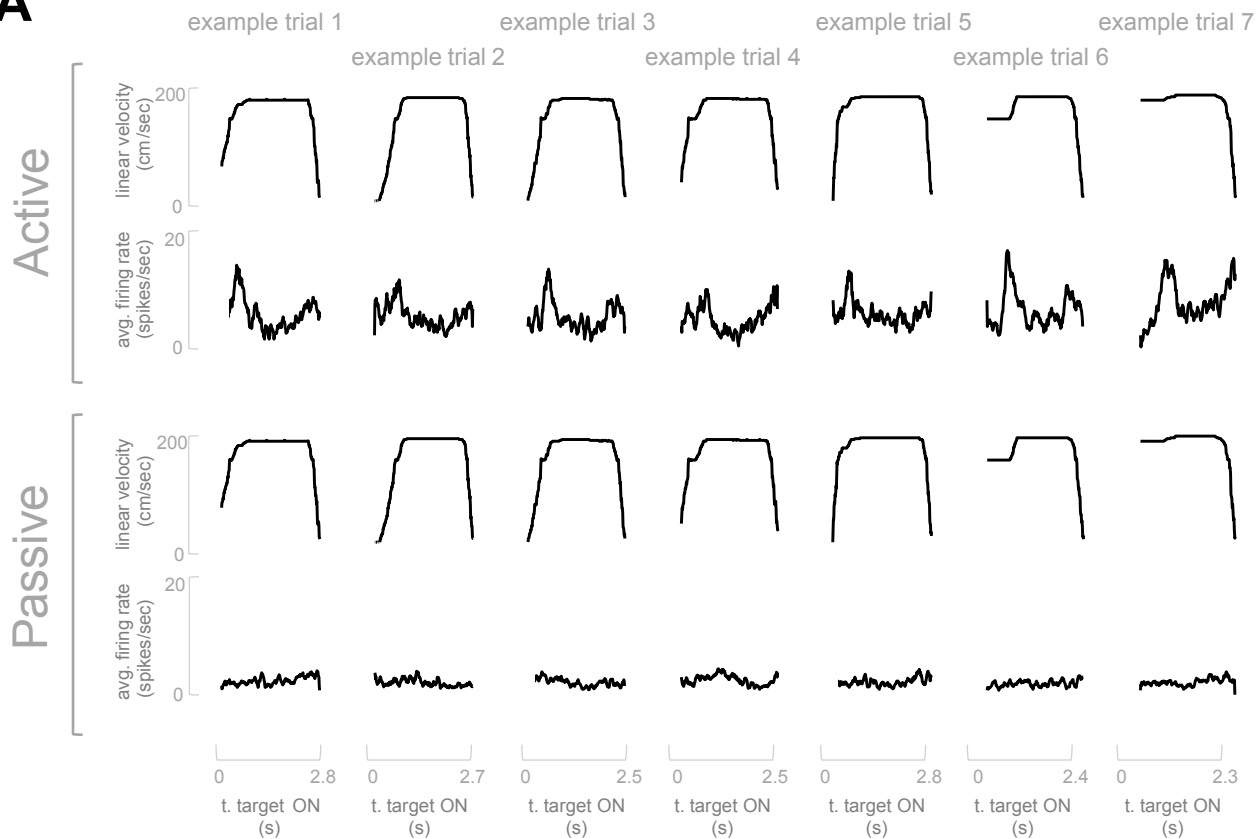**B**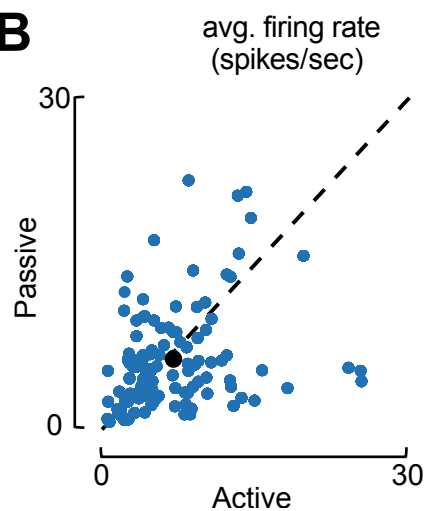**C**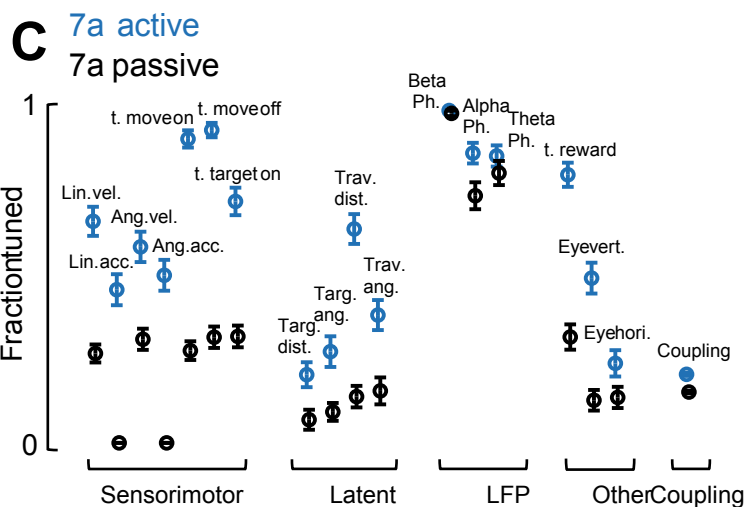

Supplement: MDAR checklist [file elife-80280-mdarchecklist1.zip › Figure2 supplement11.pdf]

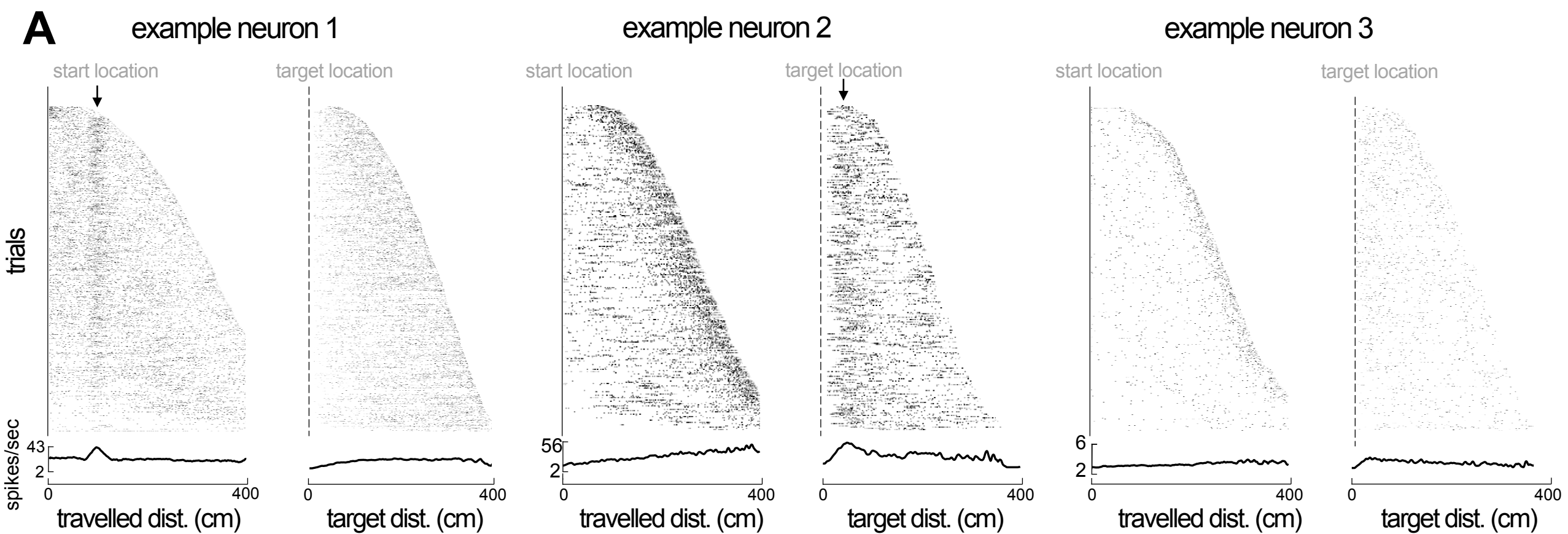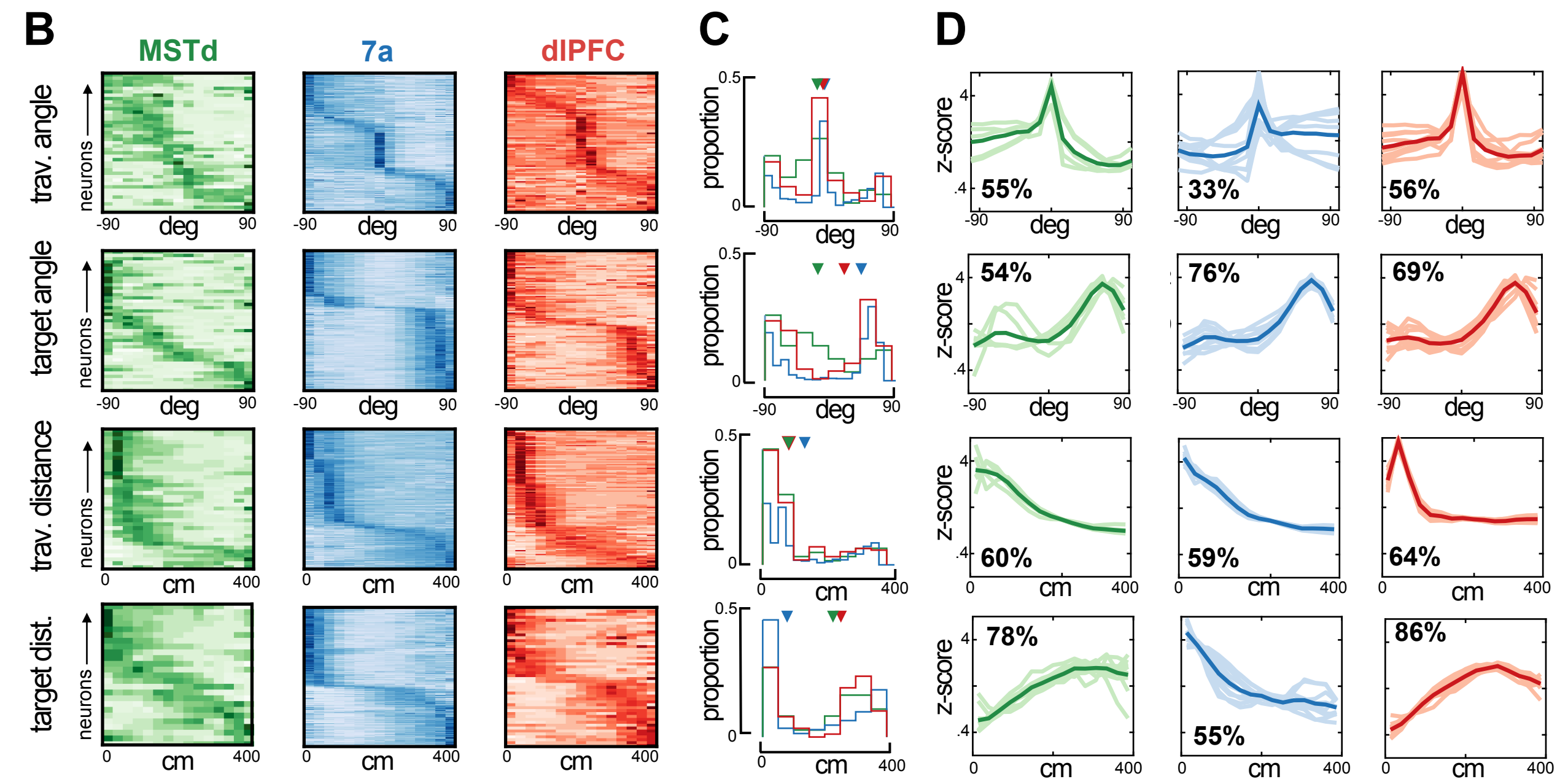

Supplement: MDAR checklist [file elife-80280-mdarchecklist1.zip › Figure3.pdf]

MSTd  
7a  
dIPFC

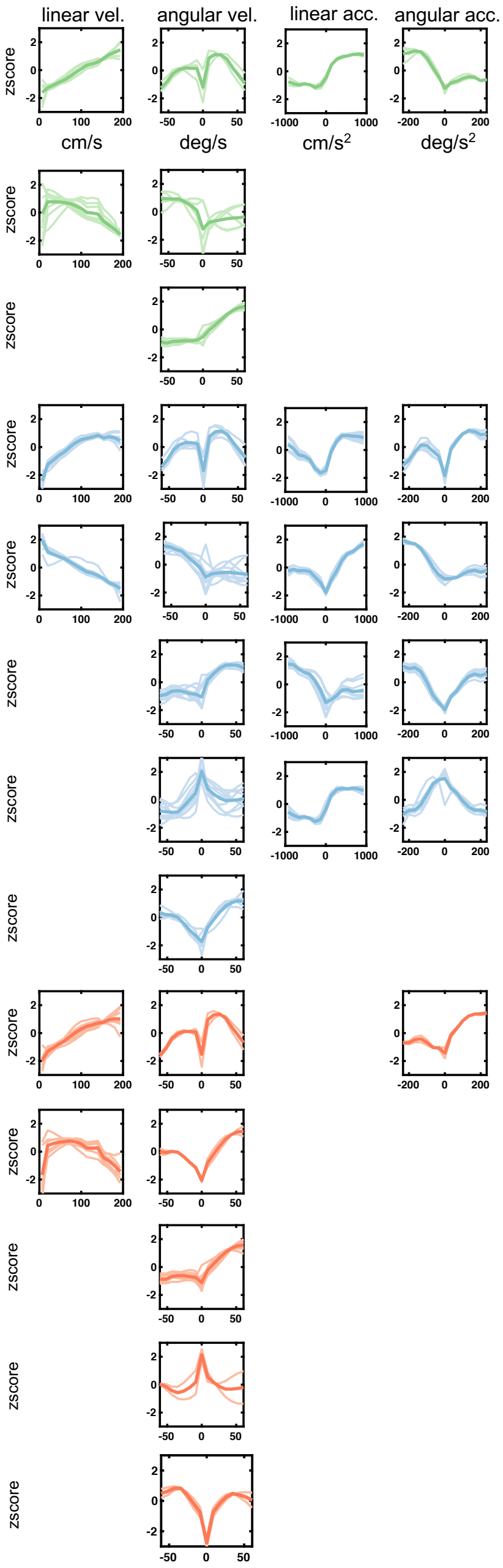

Supplement: MDAR checklist [file elife-80280-mdarchecklist1.zip › Figure3 supplement1.pdf]

MSTd  
7a  
dIPFC

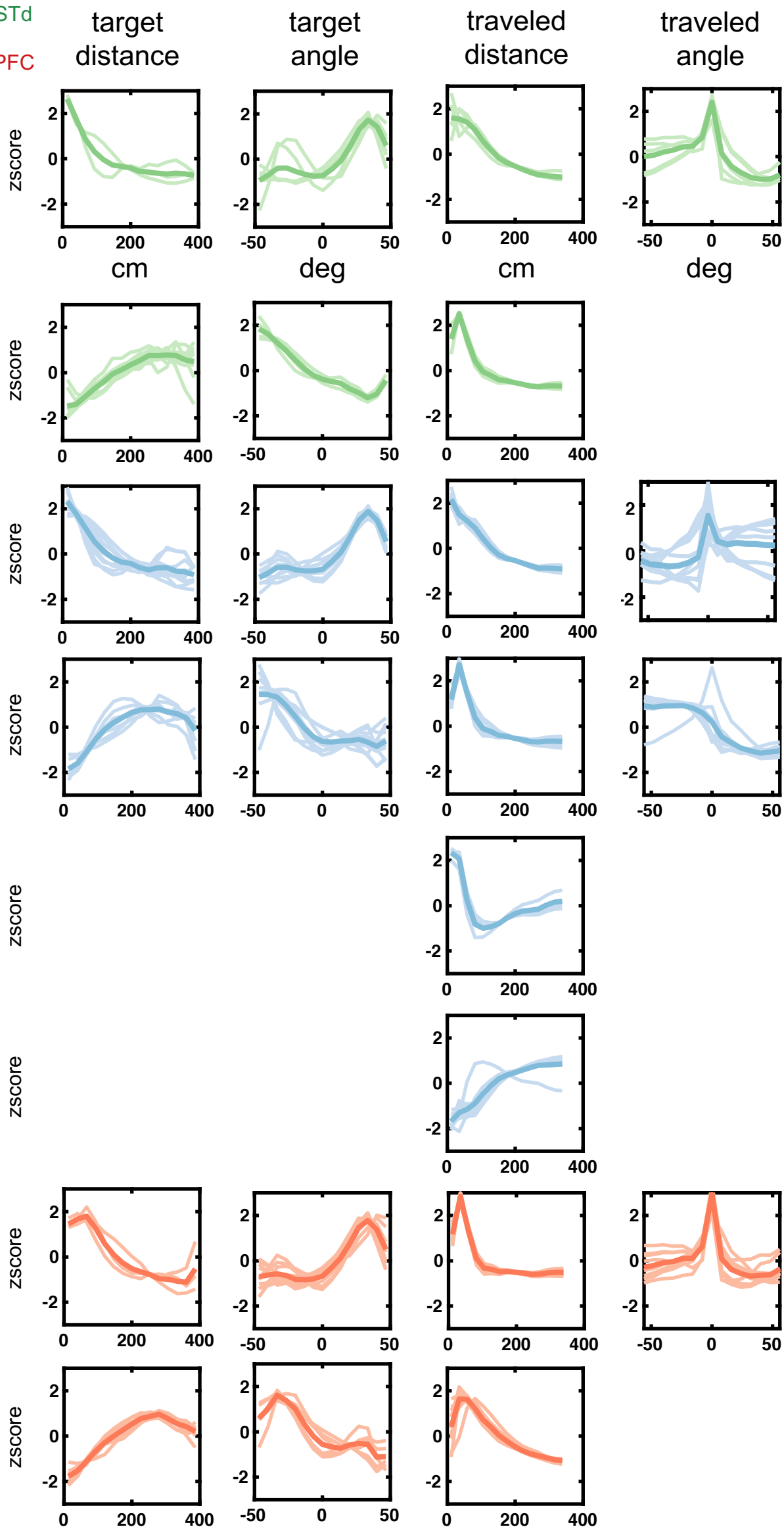

Supplement: MDAR checklist [file elife-80280-mdarchecklist1.zip › Figure3 supplement2.pdf]

dIPFC

t. reward

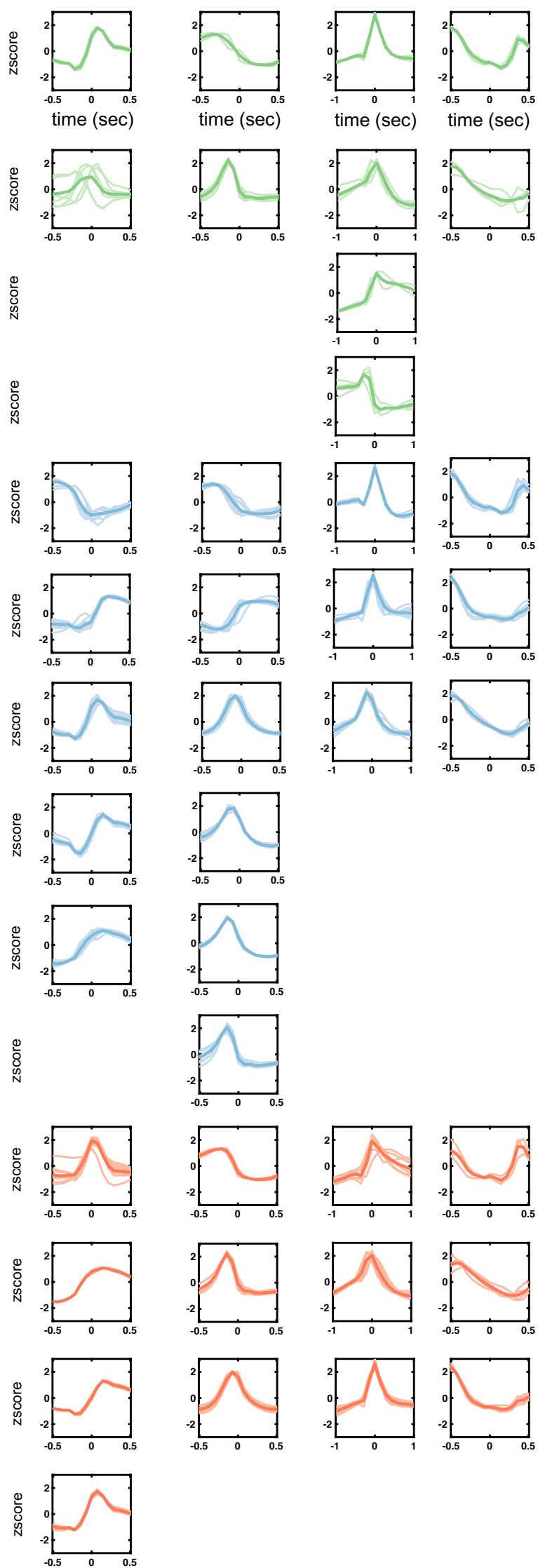

Supplement: MDAR checklist [file elife-80280-mdarchecklist1.zip › Figure3 supplement3.pdf]

MSTd

7a

dIPFC

eye  
vert.

eye  
horz.

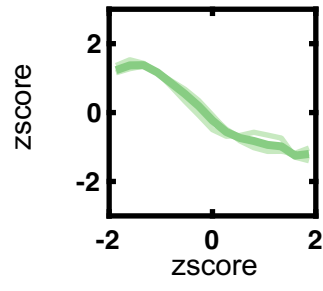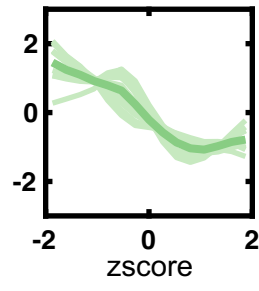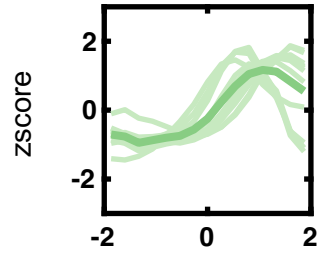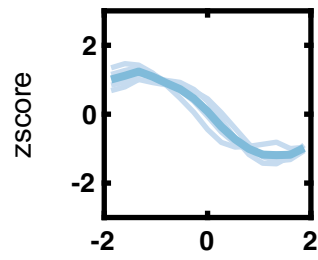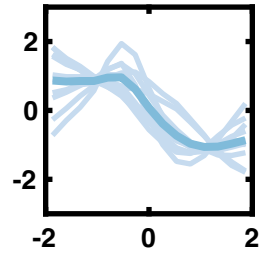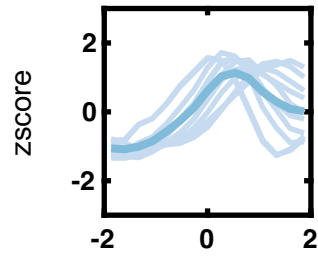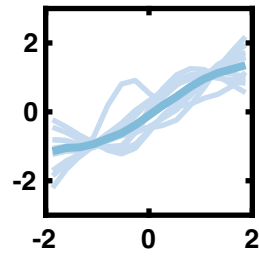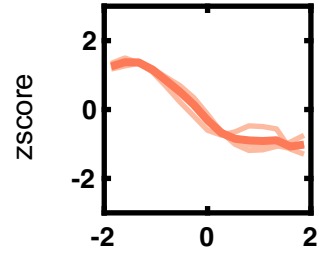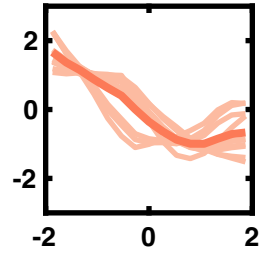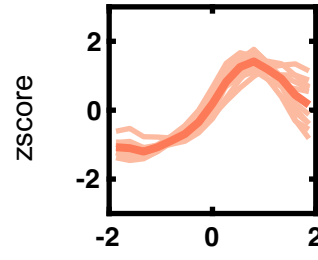

Supplement: MDAR checklist [file elife-80280-mdarchecklist1.zip › Figure3 supplement4.pdf]

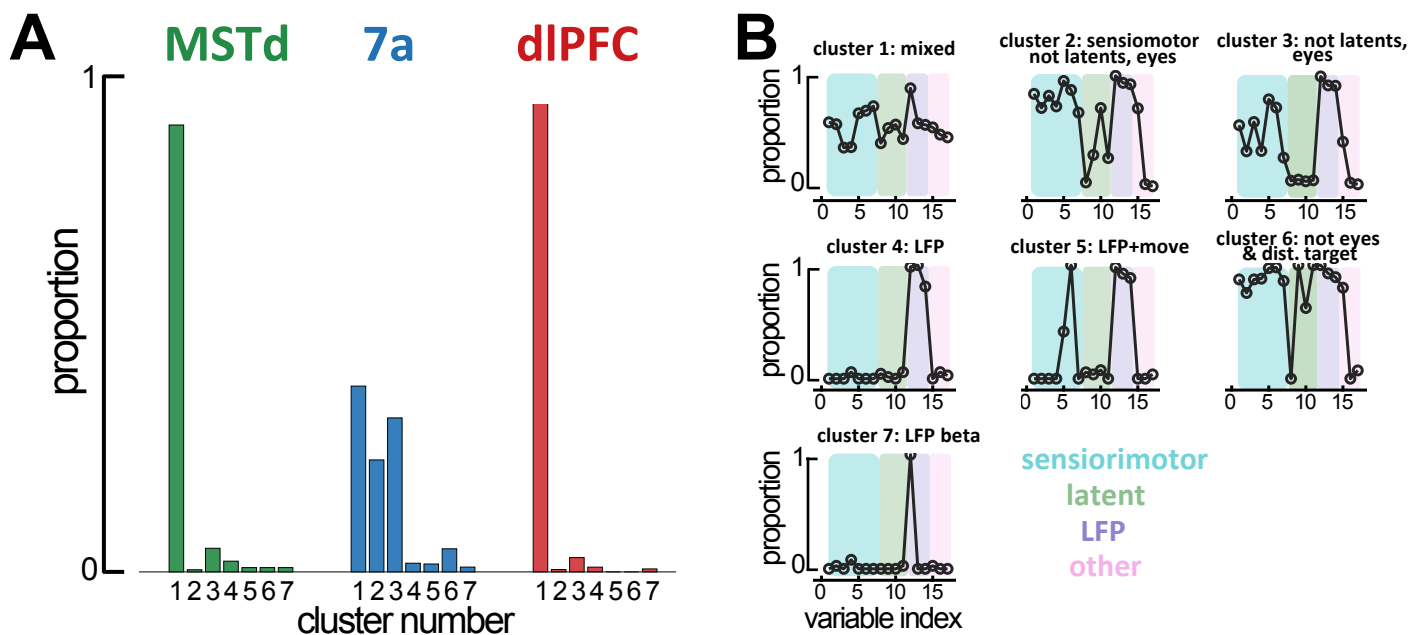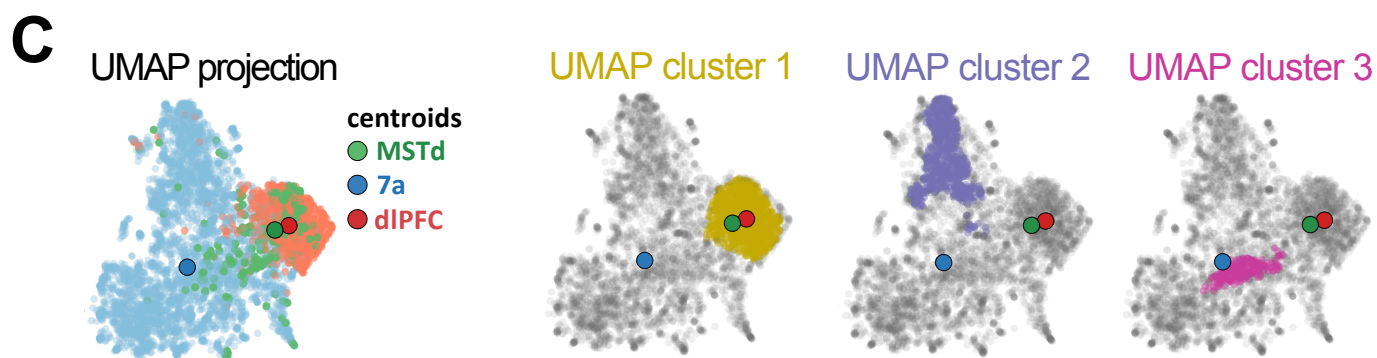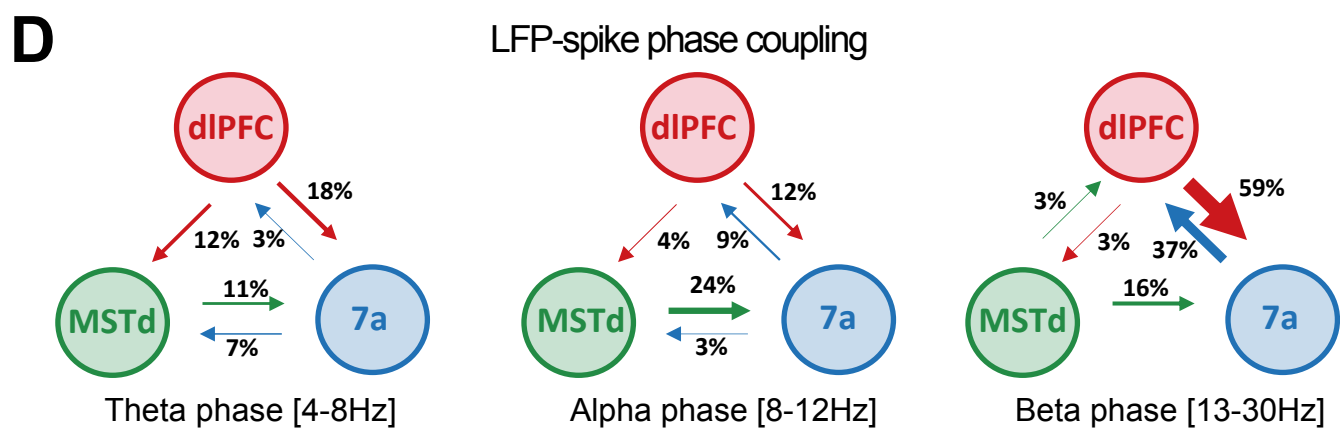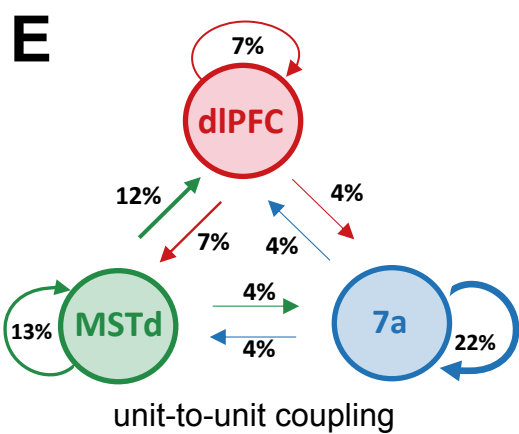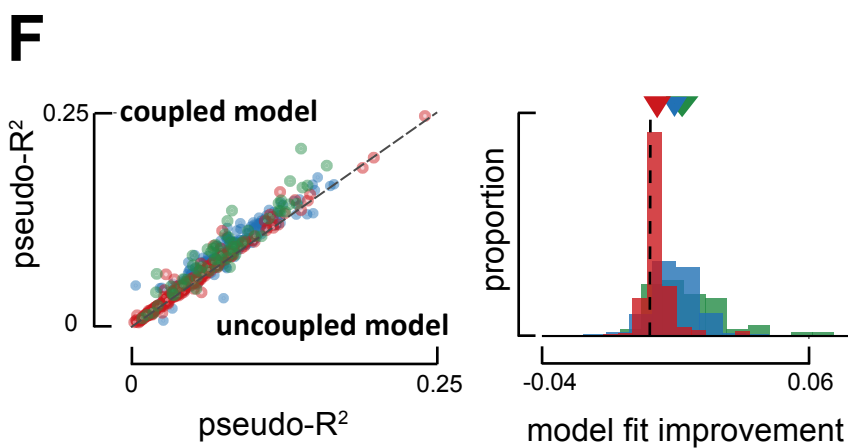

Supplement: MDAR checklist [file elife-80280-mdarchecklist1.zip › Figure4.pdf]

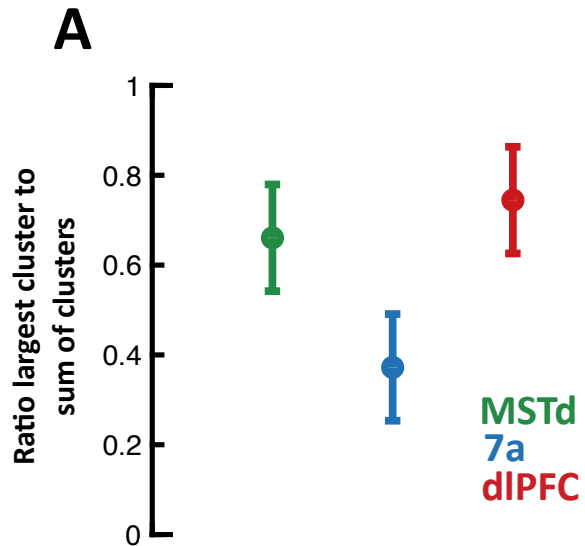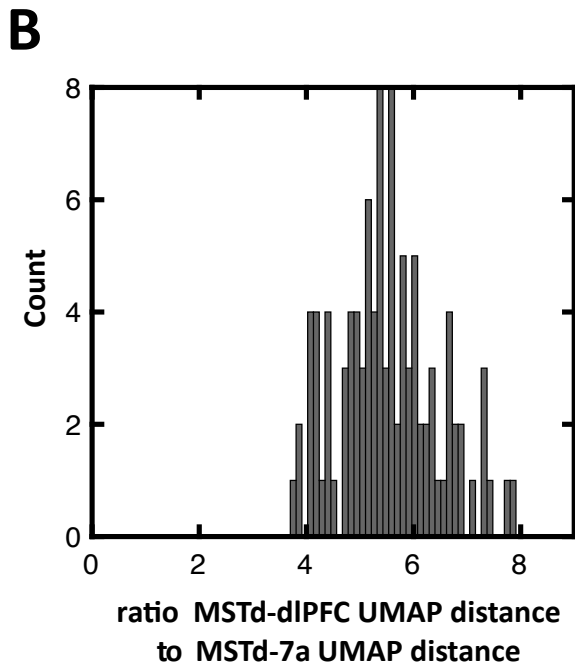

Supplement: MDAR checklist [file elife-80280-mdarchecklist1.zip › Figure4 supplement1.pdf]

sensorimotor

latent

LFP

other

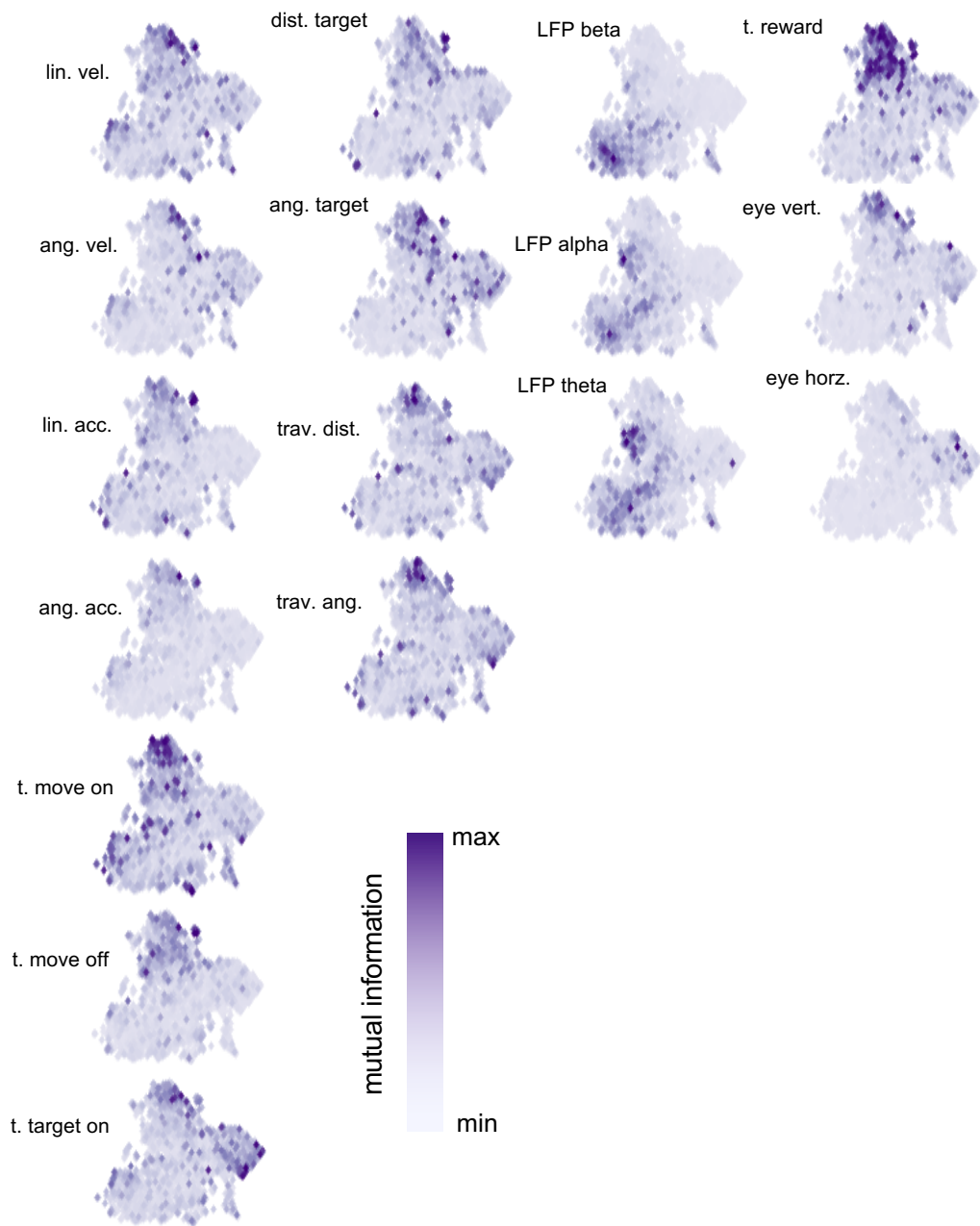

Supplement: MDAR checklist [file elife-80280-mdarchecklist1.zip › Figure4 supplement2.pdf]

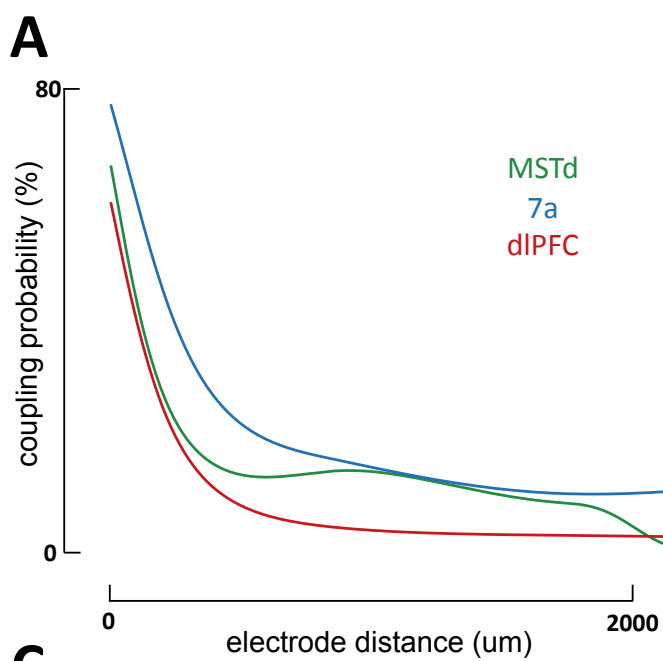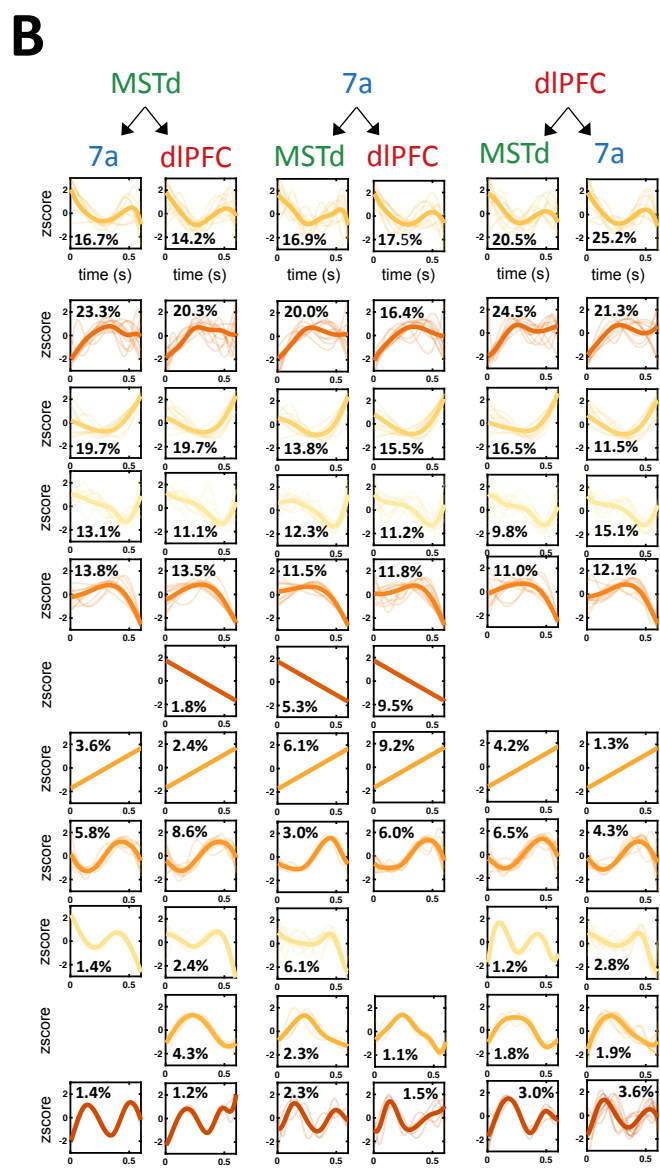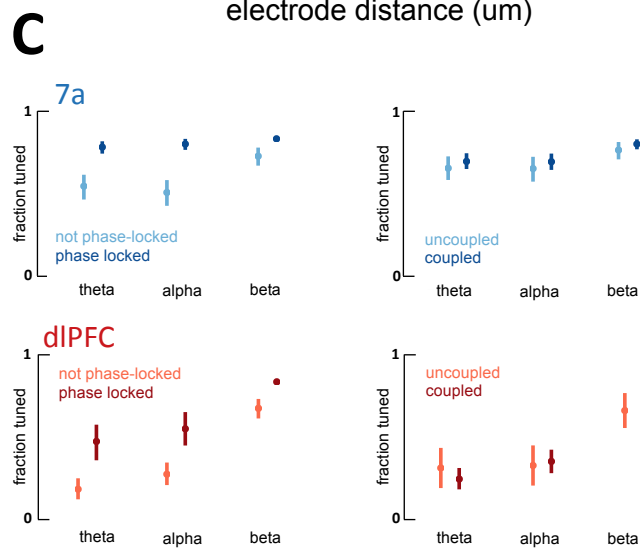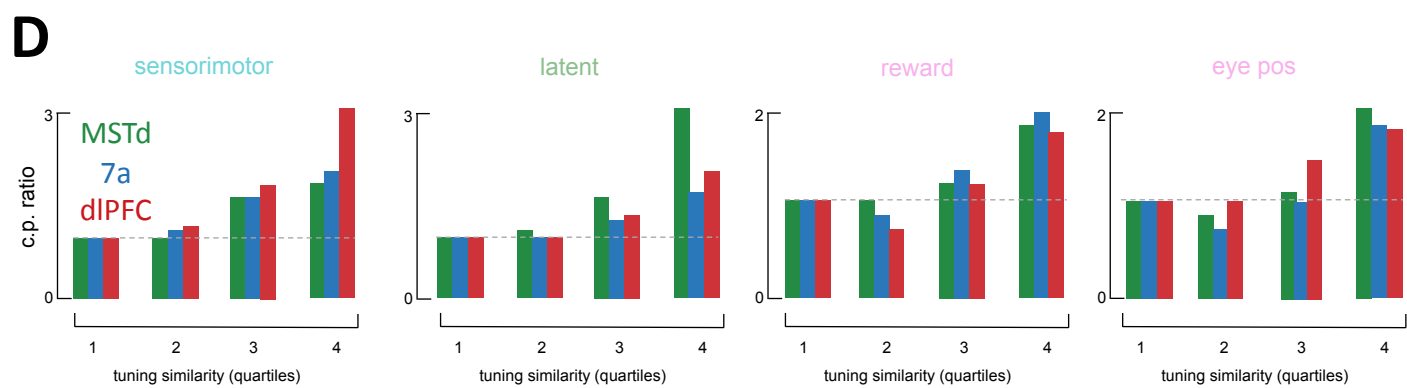

Supplement: MDAR checklist [file elife-80280-mdarchecklist1.zip › Figure4 supplement3.pdf]

**7a**

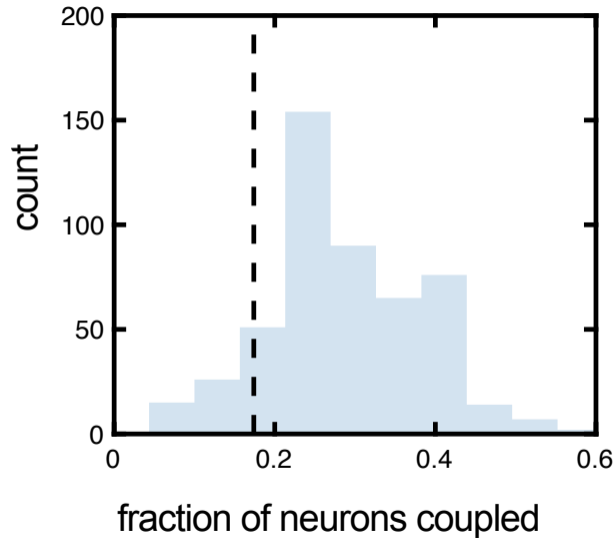

**dIPFC**

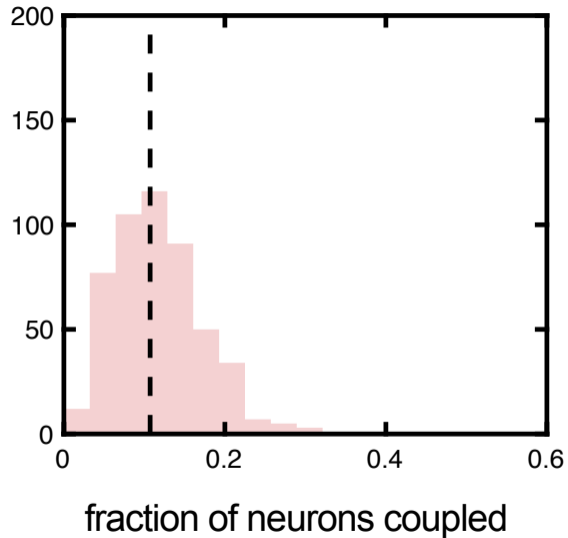

Supplement: MDAR checklist [file elife-80280-mdarchecklist1.zip › Figure4 supplement4.pdf]

**A**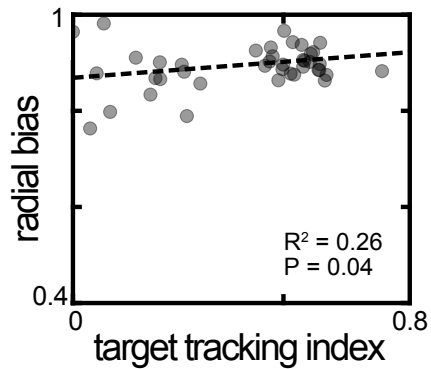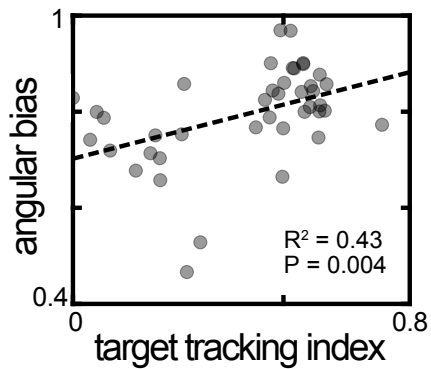**B**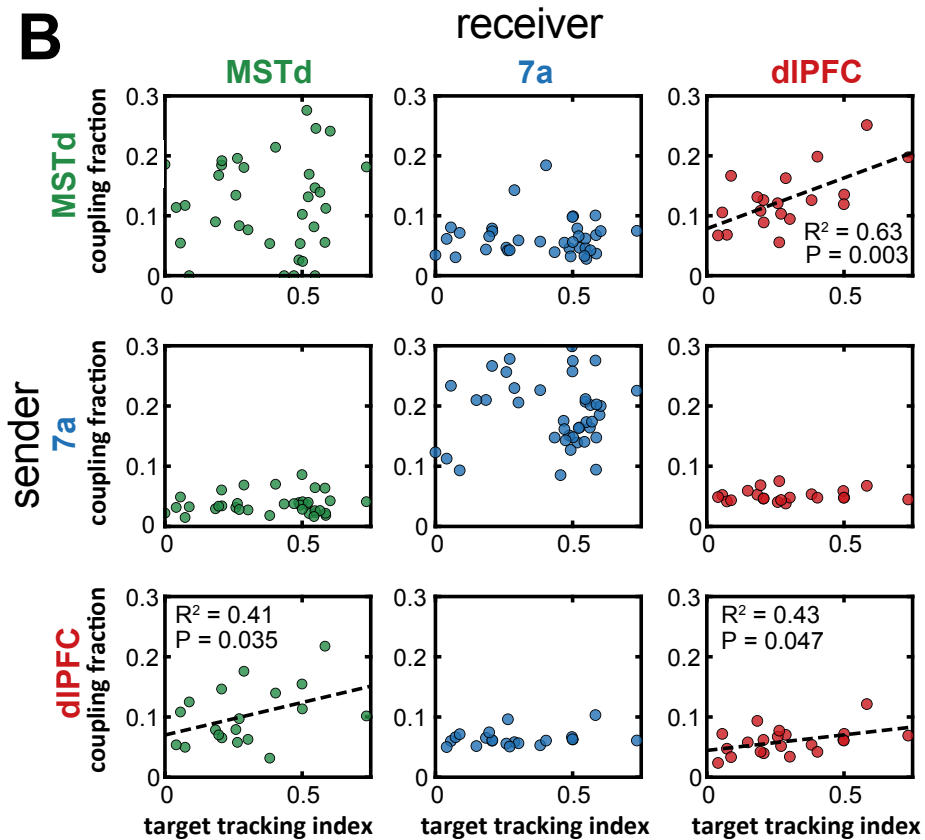

Supplement: MDAR checklist [file elife-80280-mdarchecklist1.zip › Figure5.pdf]

**A**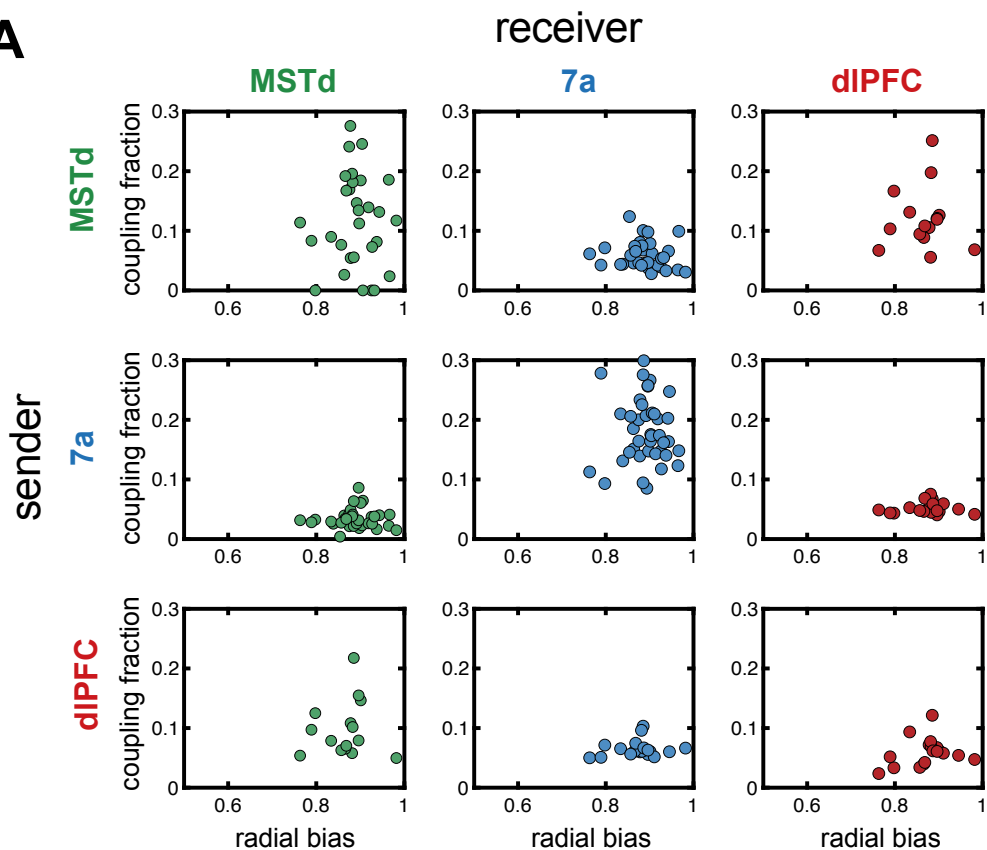**B**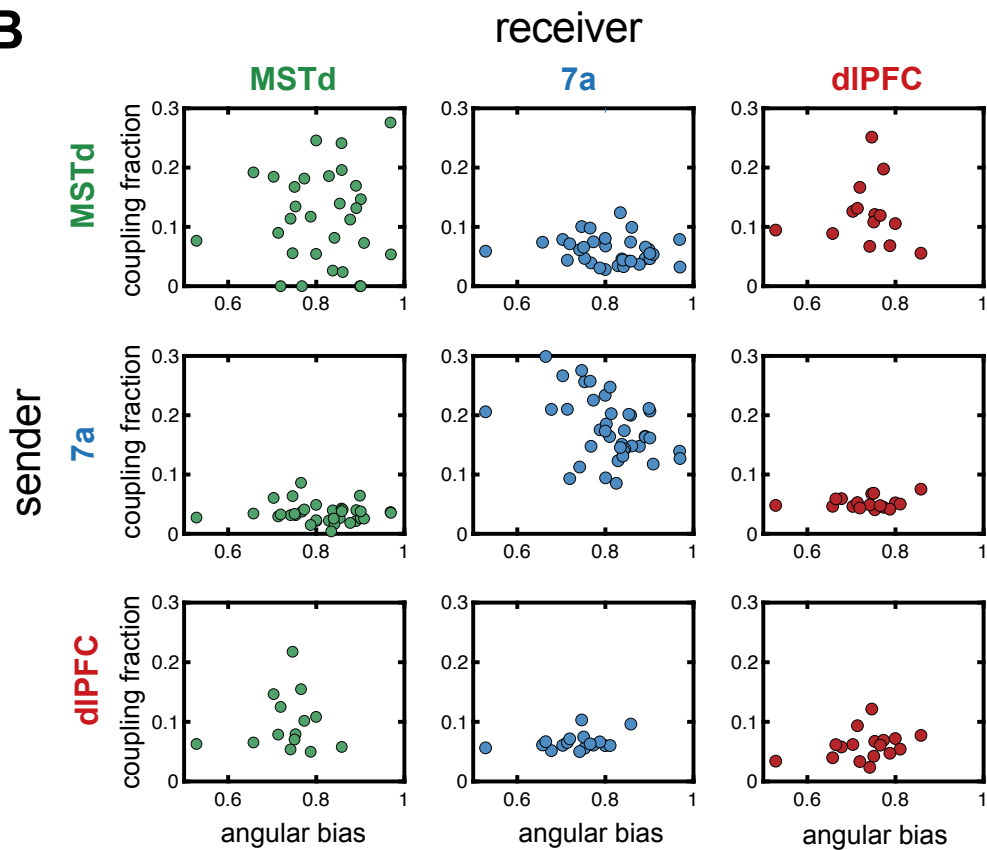

Supplement: MDAR checklist [file elife-80280-mdarchecklist1.zip › Figure5 supplement1.pdf]
